# “Emergency” multidisciplinary team meeting in cancer: a first feedback from a care team

**DOI:** 10.1186/s12967-023-04652-z

**Published:** 2023-10-30

**Authors:** Henri-Corto Stoeklé, David Billard, Jaafar Bennouna, Philippe Beuzeboc, Christian Hervé

**Affiliations:** 1 Department of Ethics and Scientific Integrity, Foch Hospital, 40 rue Worth, 92150 Suresnes, France; 2 Department of Oncology and Supportive Care, Supportive Care Center, Line Renaud Institute, Foch Hospital, 40 rue Worth, 92150 Suresnes, France; 3 Medical School, Paris Cité University, Paris, France; 4 Department of One Health and Global Health, Medical School, Versailles Saint-Quentin-en-Yvelines University (UVSQ), Montigny-le-Bretonneux, France

**To the editor**

An “emergency” multidisciplinary team meeting (MTM, or MDTM) — or "*réunion de concertation pluridisciplinaire (RCP) 'de crise*'" in French — combines some features of a hospital ethics committee with those of the MTMs, widely used in cancer [1]. More precisely, it is a new kind of MTM that integrates bioethics, as a discipline, in complex medical situations in which there is also clearly a need to identify and resolve certain “micro-bio-ethical issues” — i.e. significant tensions between medical/biological practices and moral values/standards at the level of the individual and/or the institution [2] — and to relieve “moral distress” — i.e. “the result of constraints on healthcare professional’ moral agency” [1].

In theory, we are talking here about bioethicists, human and social scientists, who join a MTM on an ad hoc basis to provide bio-ethical support, rather than the oncologists, radiologists or pathologists, for example, who seek bioethics recommendations or advice from a hospital ethics committee. Of course, an emergency MTM is not an alternative to a hospital ethics committee in terms of institutional commitment. At Foch Hospital in France, we organized an emergency MTM right from the start of the COVID-19 pandemic, but we also used this approach more recently in an extreme clinical situation in cancer.

A woman was admitted following symptoms that revealed a metastatic cancer during pregnancy. A national MTM dedicated to cancers in pregnant women proposed a therapeutic abortion in this case, to ensure that the disease could be treated as effectively as possible with the best pharmacological treatments, which are strongly contraindicated during pregnancy. The patient disagreed. She refused all treatment that might harm her unborn baby. An emergency MTM was organized by the Department of Ethics and Scientific Integrity, at the request of the Department of Oncology and Supportive Care, to help them to identify and resolve what appeared to be a micro-bio-ethical issue, and to relieve obvious moral distress.

Indeed, during this emergency MTM, we first worked together to characterize this tension between the proposal to treat the disease as effectively as possible at the expense of losing the pregnancy — i.e. a medical practice, in this case a therapeutic abortion — and the patient’s refusal of this proposal — i.e. a moral value, here, individual freedom. After a long interdisciplinary discussion, the care staff came to a consensus to administer a less effective treatment that made it possible to continue the pregnancy, based on a pragmatic bio-ethical approach balancing the far from certain survival of the patient against the almost certain happiness resulting from an ultimate, humanly legitimate project to have a child, which was accepted by the patient’s husband. This approach was approved by the hospital ethics committee at the same time. The patient was followed by the neonatal unit until conditions for a preterm birth were considered acceptable. Unfortunately, the patient died shortly after the child's birth, but the child survived and is currently in good health.

This time, we organized two subsequent meetings to obtain feedback on the bio-ethical experiences of the care staff involved soon after the event and after a longer time interval. With the approval of the institutional review board of Foch Hospital (IRB00012437), and oral informed consent from the care staff involved, we recorded and retranscribed (in French only) these two sessions. An analysis of the content of these recordings and transcriptions revealed a positive appreciation of the work of the emergency MTM, favoring “*wider dissemination*” of this approach, providing “*food for thought*” and also “*retaining the goals of the MTM*”, in other words, more collective responsibility. However, the pertinence of the name “*MTM*” was also discussed, due to the “*more ethical than purely medical*” reflections of an oncological and/or supportive care MTM.

At this stage, we should add a more practical explanation of this choice of name and model. In our experience, an “ethics committee” — a “clinical ethics consultation”, etc — can have the disadvantage of eliciting a certain mistrust among the care staff. This mistrust can destroy the sometimes necessary interactions between bioethicists, human and social scientists, and the care departments, units or teams, as in this extreme clinical situation, particularly if the care staff concerned are managers. We therefore hypothesized that using a name and model similar to that of a medical structure that most care staff consider to be essential, especially in cancer, would consolidate such interactions in the context of bioethics, although it should again be stressed that an emergency MTM is not an alternative to a hospital ethics committee in terms of institutional commitment. It acts as an intermediary between the care staff and the hospital ethics committee.

In conclusion, the objective of this preliminary work is to raise the possibility of the implementation of an emergency MTM to improve the identification and resolution of micro-bio-ethical issues in major health crises and/or extreme clinical situations, in cancer, and to relieve moral distress. One perspective would involve activating and validating this system in other cases, or even other hospitals, to confirm, in parallel, the suitability of this model and its name, in cancer, and perhaps other diseases.

## Data Availability

The datasets generated and/or analyzed in this current study are not publicly available because they are entirely in French, but are available from the corresponding author on reasonable request.
